# Cardiologists’ perceptions on multidisciplinary collaboration in heart failure care - a qualitative study

**DOI:** 10.1186/s12913-021-06179-9

**Published:** 2021-02-23

**Authors:** Willem Raat, Miek Smeets, Isolde Vandewal, Lien Broekx, Sanne Peters, Stefan Janssens, Bert Vaes, Bert Aertgeerts

**Affiliations:** 1 Department of Public Health and Primary Care, KU Leuven, Kapucijnenvoer 33, Blok J, Bus 7001, 3000 Leuven, Belgium; 2 Ebpracticenet, Leuven, Belgium; 3 Department of Cardiovascular Diseases, Universitaire Ziekenhuizen Leuven, KU Leuven, Leuven, Belgium

**Keywords:** Heart failure, Cardiology, Multidisciplinary, Collaboration, Perception

## Abstract

**Background:**

Cardiologists play a key role in multidisciplinary care by guiding heart failure (HF) management in the hospital and in the community. Regional implementation of multidisciplinary health care interventions depends on how they perceive collaboration with other health care disciplines, yet research on this topic is limited. This study aimed to explore the views and opinions of cardiologists on multidisciplinary collaboration in HF care.

**Methods:**

We conducted a qualitative study based on face-to-face semi-structured interviews with 11 Belgian cardiologists between September 2019 and February 2020. We used the Qualitative Analysis Guide of Leuven (QUAGOL) method as guidance for data analysis until data saturation was reached.

**Results:**

Cardiologists consider the general practitioner (GP) and HF nurse as the most important partners in HF management. Cardiologists identified four problems in current multidisciplinary collaboration: the communication of a HF diagnosis to the patient, advanced care planning, titration of HF medication by the GP and electronic data exchange and communication. Three themes emerged as ideas for improvement of HF care: 1) expansion of the role of the HF nurse, 2) implementation of a structured, patient-centered, and flexible model of disease management program and 3) integrated data approaches.

**Conclusion:**

Cardiologists value close cooperation with GPs in HF management. They advocate an expanded future role for the HF nurse, increased eHealth, and structured disease management to optimize current HF care.

**Supplementary Information:**

The online version contains supplementary material available at 10.1186/s12913-021-06179-9.

## Background

Heart failure (HF) is an important and growing public health problem that affects millions of patients worldwide and has enormous impact on patients’ quality of life and global health expenditures [1]. The prevalence of HF is substantially higher in older patient groups, and most patients suffer from one or more chronic conditions [2]. Consequently, health care for the HF population generally takes place across different settings and health disciplines. The goal of HF management is to provide a seamless system of care that embraces both the community and hospital throughout the health care journey [3]. Multidisciplinary team management and cross-setting coordination are fundamental to this approach and demonstrated substantial reductions in hospital admissions and mortality when integrated into coherent disease management programs (DMPs) [4, 5].

Cardiologists, HF specialist nurses and general practitioners (GPs) are key members of the multidisciplinary HF team [6]. The successful implementation of DMPs in contemporary practice depends on these health care providers’ attitudes and perceptions regarding multidisciplinary collaboration. Previous research therefore elucidated the views of GPs and HF specialist nurses on interdisciplinary care [7, 8]. Past exploration of cardiologists’ perceptions on community care [9] and cooperation with HF nurses [7] shed light on the importance of the HF nurse in patient and team communication and the challenges in ensuring efficient and consistent transition from acute care settings to the community. However, to our knowledge, the opinions of cardiologists on cooperation with GPs remain uninvestigated.

The objective of our study was therefore to explore cardiologists’ perceptions on cooperation with GPs and multidisciplinary collaboration in HF care.

## Methods

### Design

We opted for an exploratory qualitative design to study cardiologists’ perceptions. We took a phenomenological approach [10] and conducted face-to-face semi-structured interviews. The consolidated criteria for reporting qualitative research (COREQ) checklist was used to report our findings [11].

### Participants and recruitment

We asked cardiologists and residents from different hospitals in the Belgian province of Limburg to participate. Residents had to have had at least 4 months working experience in a cardiology department. We contacted eleven cardiology departments by phone and email. One month after the initial contact, we sent a reminder via a personal email. Finally, we contacted cardiologists by phone to voluntarily participate without remuneration for participation in the study. We aimed to balance gender, type of hospital (secondary, tertiary), cardiology subspecialty (HF vs general) and years of experience (purposive sampling). Belgian health care services are financed by proportional social security contributions and progressive direct taxation. There is compulsory health insurance combined with a mostly private system of health care delivery, based on independent medical practice, free choice of physician and predominantly fee-for-service payment [12]. There is no national HF strategy, nor funding for specialized HF nurses. A national hospital accreditation program for HF care exists, but its impact on HF care and outcomes is limited [13].

### Data collection

Two of the authors (IV and LB) conducted the face-to-face semi-structured interviews between September 2019 and February 2020. One of the authors functioned as interviewer, the other as observer taking notes. We chose this approach to maximize the familiarity of researchers with each interview and context. Both researchers are female and worked as GP trainees in the Belgian province of Limburg at the time of the interviews. They were familiar with some of the interviewees within a professional context. SP trained both authors in conducting interviews. The interviews took place in the participating cardiologists’ hospital or consultation office. We used a topic list that was based on the literature and discussions between the authors to structure the interviews (supplemental file 1: Topic list). Two test interviews helped to refine the interview questions and allowed IV and LB to improve their interview technique.

All interviews were conducted in Dutch, the native language of the interviewers and cardiologists, and audio recorded. A professional language editor translated the interviews. We conducted interviews until we reached data saturation [14]. We defined data saturation as the moment when the two previous interviews no longer contributed any new elements and when a certain category had been exhaustively described in all its dimensions and variations. This signified that additional interviews would no longer provide new insights.

### Data analysis

All interviews were verbatim transcribed, including both verbal and non-verbal signs (conversation analysis) [15]. We adhered to the principles of the Qualitative Analysis Guide of Leuven (QUAGOL) as guidance in data analysis [16]. The procedure consisted of two steps: 1) a preparatory phase using word processing software and 2) the actual coding process, facilitated by NVIVO 12 software (QSR International, Melbourne Australia).

The aim of the preparatory phase was to become as familiar as possible with the interview data in order to compile a list of concepts as starting point for the coding in NVIVO 12. First, two authors (LB and IV) captured the context and essence of each interview separately and then discussed this together. Afterwards, a conceptual scheme was drafted for each interview. The usability of this scheme was monitored by repeated comparisons with the interviews. Finally, the research team (WR, LB, IV, MS, and SP) analyzed and compared the conceptual schemes of the various interviews to compile a list of concepts.

Two authors (LB and IV) performed the coding process using NVIVO 12 software. First, they coded the data by linking each fragment of text to one of the concepts in the list. Consequently, LB and IV generated an initial coding tree in the program. The relevance and usefulness of the codes and concepts were evaluated by the research team (WR, LB, IV, MS, and SP) and adjusted when necessary. In the final step, three researchers (WR, LB and IV) separately extracted the essential storyline from the data. After reaching consensus, the findings were discussed within the entire research team.

We used investigator triangulation in a group of diverse researchers across all stages of the analytical process to increase the reliability of our results.

## Results

### Participants and recruitment

We contacted 41 cardiologists by email. Twelve cardiologists agreed to participate in our study (Table 1). Reasons to refuse participation were lack of time, lack of experience or previous participation in GP trainee research. Lockdown measures caused by the SARS-CoV-2 virus prevented one interview from taking place. However, we obtained a large variation of practice types and the cardiologists varied by age, years of experience and fields of interest within cardiology. The interviews lasted between 24 and 87 min.

**Table 1:** Participant characteristics

ID	Sex	Years of experience (range)	Type of hospital	Field of interest within cardiology
C#1	Male	Junior resident	Tertiary	/
C#2	Male	25–30	Secondary	Prevention and cardiac revalidation, including heart failure
C#3	Male	10–15	Secondary	Heart failure, intensive care and cardiac devices
C#4	Female	Senior resident	Tertiary	General cardiology, heart failure
C#5	Male	20–25	Private practice Secondary	General cardiology
C#6	Female	0–5	Secondary	Echocardiography, cardiac revalidation and heart failure
C#7	Male	10–15	Primary	General cardiology
C#8	Male	5–10	Tertiary	Heart failure and intensive cardiac care
C#9	Female	0–5	Secondary	Heart failure, imaging and intensive care
C#10	Male	5–10	Secondary	Heart failure, imaging and echocardiography
C#11	Female	15–20	Secondary	Cardiac revalidation

### Theme 1 – interdisciplinary collaboration

Participants generally declared themselves, GPs, and specialized HF nurses as the most important disciplines in the multidisciplinary HF team.

#### Cardiologists’ role in HF care

Participants described their role in diagnosing HF as dual. First, they established the diagnosis and etiology of HF by a thorough medical history, clinical examination and echocardiography. Second, they were co-responsible for communicating the diagnosis to both patient and GP. The proper communication of a HF diagnosis remained problematic, both to patients and GPs.

*“There are many people with HF who are not even aware of the diagnosis. “Oh, do I have a heart problem?” “Yes, you’ve had it for 7 years now.”, “Nobody has ever told me that.” I think the cardiologist, at the very least, should communicate it. However, if it wasn’t clear for the patient during the consultation, I think the GP could mention it in the next visit. For instance: “I saw the report from the cardiologist you went to the other day: you really have a heart problem”.” – C6*

*“Perhaps, for us, it is also important to put it clearly in the discharge summary to the GP: ‘Patient has HF with reduced ejection fraction’. If the letter states ‘hypertrophic cardiomyopathy’ instead, I don’t know if that alerts GPs to be mindful of the problem.” – C6*

They reported their most important role in managing HF is the strategic planning of the treatment objectives, as well as the supervision of management in primary care.

On the topic of advanced care planning and end-of-life care, participants noted a lack of structured advanced care planning in HF patients, reinforced by the oscillation and unpredictability of HF disease progression.

*“It is a problem to find a good time to bring it up. You can’t just rattle it off in two minutes. The acute phase is not the right moment either. (...) If you look at the curve of HF patients (makes a downward, wavy curve with his hands), at which point in time should you do this? It’s not straightforward, and I find it this the hardest thing to do.” – C11*

#### GPs’ role in HF care

Participants commented that GPs perform three vital roles in a multidisciplinary HF approach.

First, the timely interception of a possible HF diagnosis and signs of decompensation. Frequent follow-up, elaborate history taking, and thorough clinical examination are fundaments of this sentinel function. They generally viewed natriuretic peptide (NT-proBNP) testing as an important aid in the diagnosis.

*“As a GP, if you suspect HF, then you have to rule it out and refer them. If you don’t diagnose it, they just keep going, symptoms and all. With on-time referral, it’s possible to avoid hospitalizations and problems.” – C9*

Second, they stressed the importance of the GP in assessment of compliance and motivation towards behavioral change. They regarded this as specifically important because of the close connection between patient and GP.

*“Most often, they’ve known the patient longer than we do. They know their environment, home situation, family context and such much better than we do. And obviously all this will also play a role besides the purely medical aspect.” – C8*

Third, they saw the GP as essential in executing treatment objectives and follow-up in primary care.

*“The GP also has to manage and realize the strategy, provide the follow-up, a lot of times titrate medication – the aspect we’ve just discussed - and recognize and interpret new problems. I think it’s very broad.” – C3*

*“HF specifically often requires continuous monitoring, also in home settings. And we are not going to do that. The minute they are gone, out of the hospital, well … your patient might, think about your advice for a few weeks, but you actually need some sort of continuous follow-up. That is precisely the role of the GP.” – C1*

After discharge for a HF hospitalization, participants found that close follow-up by the GP is crucial in preventing early readmission.

*“Frequently, diuretics need to be reduced, ACE inhibitors and beta-blockers need to be titrated upwards. So, this first phase after hospitalization is a very unstable phase. I think this phase needs to be managed by GPs as well as by specialists, in some kind of collaborative manner.” – C10*

All participants agreed that frequency and setting of follow-up in outpatient care is to be individualized for every patient. They feel that patients with stable HF can be followed-up in primary care with a yearly cardiology appointment. Patients with more complex conditions need tailored followed-up.

*“Once people are on track, and they’re doing well, then there is no reason for those people to consult us too much. Then it’s more a condition that needs to be followed up by GPs.” – C7*

Respondents were complimentary on some areas of pharmaceutical HF management by GPs such as flexible diuretic use to treat congestion. They noted, however, that GPs were often reluctant to titrate HF medication such as beta-blockers, ACE-inhibitors or sacubitril-valsartan, primarily because of fear of side effects. Because of this hesitance, the participants often monitored titration themselves.

*“Upward titrating of HF medications, it hardly ever happens. You’ve probably heard that one before … And I kind of get it.” – C8*

#### HF nurse role in HF care

Several participating cardiologists reported that HF nurses offer a vital support structure in the hospital and liaison with outpatient health care.

*“Our consultation schedule is fully booked. At the moment, two parallel consultations take place at the same time and we hop over, from one to the other. So they do all the preparatory work, which is a lot, and we join the consultation at the end.” – C11*

*“The HF nurse is a liaison between the patient and actually both doctors, the cardiologist as well as the GP.” – C7*

The main benefit of the HF nurses is availability. HF nurses have more time to focus on patient motivation and education. This also provides them with a deeper understanding of patients’ background.

*“They also participate during the consultation. They make sure the medication is explained once more, walk the patients through the medication list … so, the extra time a doctor usually lacks, they can provide that for the patient.” – C8*

*“They know the patients very well. They know their family situation and their background, so I think specialized nurses are an enormous asset in quality patient care.” – C6*

Some cardiologists pointed out that GPs preferred to contact cardiologists directly, rather than through the HF nurse. They viewed this hesitance as understandable but unfounded, given the experience and HF-specific knowledge of HF nurses.

*“We have also noticed that HF nurses are actually not often contacted by GPs. However, most of the time they can actually help GPs. (...) I don’t know whether it’s some sort of perception that ‘it’s only a nurse’, because we in fact HF nurses have had extensive extra training and are concerned with nothing other than HF. They know the guidelines better than … well you, me, some of the other cardiologists.” – C6*

#### Organizing care across health care settings

Throughout all interviews participants perceived proper communication as quintessential for good collaboration. They discussed various channels as a means for communication.

First, they acknowledged the importance of a good discharge letter to establish proper follow-up in the community.

*“Concerning patients, I think the most important thing still is and will always be the discharge letter. And making sure it holds all the information the GP needs to know.” – C6*

Moreover, they stressed the importance of clearly communicating relevant information to GPs, while also expressing a fear of overburdening GPs with information.

*“Reports of colleagues that mention an ejection fraction buried somewhere between the lines and not explicitly in their conclusion, while you can expect that the GP will only read the conclusion. I don’t know if GPs read much else other than the conclusion in everyday practice, to be honest … ” – C6*

*“GPs get 10 to 20 letters a day with patient information from all kinds of specialists. How do you make sure follow-up goes well? How do you make sure that people don’t fall through the cracks?” – C2*

Second, participating cardiologists regarded email or telephone as a useful tool for providing advice to GPs when contacted, mostly questions concerning patients with deteriorating clinical conditions. Some centers created a special telephone service or mailing address to facilitate access and instruction.

*“All GPs have my email address, and they know I usually respond within a couple of days. And if it is urgent, they can always phone.” – C7*

*“We also have a ‘doctor-line’: a phone number that is only accessible for GPs, from 8 AM to 8 PM. Its purpose is to be available to a GP in need of an urgent consultation, for whatever reason (a patient consultation, consultant’s advice or whatever). (...) So for us, that’s a way not to be constantly disturbed for appointments and such.” – C2*

Third, they reported conflicting experiences regarding eHealth tools and electronic platforms or hubs facilitating communication between electronic health records (EHR).

*“It used to be so that we didn’t know which medications they were actually taking, what were the last lab results or how did the last ultrasound in the hospital go... Nowadays, we can see everything from everyone and that makes it easier.” – C5*

*“Because right now, it doesn’t not work half of the time and I find the hub such a clutter of reports. You just can’t find your way in it. (...) Considering what the hub looks like now … I think it’s horrible. It just isn’t organized.” – C6*

### Theme II – a vision on quality improvement

#### Digital tools

Participants considered increased digitalization of patient information a possible tool for standardizing and improving patient care as well as facilitating communication.

*“Every person that is involved in taking care of a patient needs to have access to the data of that patient. I believe that if you store more data digitally, you will have a better view on the patient and you can take better care of him. Digitalization is a necessity.” – C7*

In addition, they hypothesized that increasing digital support and platforms could facilitate telemonitoring of parameters and eventually, teleconsultation.

*“There will be devices, smartwatches and other systems for measuring parameters. Once patients are linked to the HF clinic, these parameters will be followed pro-actively.” – C5*

They noted three important prohibitive factors for increased digitalization: cost, patient age, and problems with the current digital infrastructure.

*“They are mostly elderly people! I mean, there are some younger patients, but you cannot ask these elderly patients to use an application on a mobile phone and register their blood pressure.” – C11*

#### Cardiologist – GP colloquium

Several participants saw improved continuing medical education (CME) as an important tool for quality improvement, either via social media or in person. They deemed CME to be an important instrument to foster physicians’ knowledge and sustain local interdisciplinary social networks.

*“Speaking at a GP gathering occasionally … that’s a way to get the GPs on board. And also, a chance to listen: is there anything from your point of view that needs improving? What bothers you in our way of working? What isn’t working? You can provide a listening ear, see if they would like to change anything. (...) Giving a talk is always useful to find out what is going on.” – C3*

Other participants proposed the idea of a multidisciplinary contact for HF, analogous to the multidisciplinary consultation for cancer, in which several disciplines jointly discuss complex HF patients’ treatment plans.

*“Comprehensive care in HF is non-existent. There is room for improvement (...) maybe like a multidisciplinary oncological consultation. A meeting where we discuss the diagnosis, the treatment plan (...) This would lead to more attention to the overall treatment plan for a particular patient.” – C8*

#### Structural support for multidisciplinary HF care

All participants valued an expansion of the role of the HF nurse as essential in a team-based approach of HF.

*“In the future, I think HF nurses, under strict instructions and after extra training, could initiate medication, titrate and reduce medication and take blood tests. Obviously, there need to be some changes in health care first to make this possible, but I think it’s the way to go.” – C2*

They saw the HF nurse as an important driver for integrated care. Many participants believed in a role for HF nurses as a liaison and support for primary care. They also saw opportunities for the integration of specialized HF nurses in outpatient settings.

*“They are definitely going to play a part in the integration of all HF care settings (...) I would call them facilitating factors for collaboration between all settings: the hospital, the patient's home and primary care. I think they will be an important factor.” – C8*

Several respondents noted the financial burden of employing HF nurses. They unanimously called for structural funding for HF nurses in the hospital.

*“In my opinion, the use of specialized HF nurses is essential in DMPs. I think these programs will lead to a decline in hospitalization rates, which will reduce health care costs. Therefore, there should be financial to recruit HF nurses in HF care teams.” – C7*

Finally, throughout all interviews, participating cardiologists expressed the hope that increased funding and personnel for HF care would increase personal time with their patients.

*“The most important thing is that you should take time. Those patients need lots of attention and time and someone has to give that to them.” – C11*

They saw disease management programs or HF care pathways as an instrument to guarantee structured follow-up for the patient but were apprehensive of having to adhere to a rigid framework.

*“I don’t think we can stop that. It’s inevitable. But I think these standardized care trajects and protocols should always be adjusted to the individual patient. I don’t think it could work any other way.” – C7*

## Discussion

In this study we investigated perceptions of cardiologists on multidisciplinary HF care and collaboration with general practitioners. Participating cardiologists identified the HF nurse and GP as crucial partners in multidisciplinary HF care and recognized a dual role for GPs: first, the timely interception of possible HF diagnoses and signs of decompensation and second, the integrated follow-up of patients in the home setting, with close follow-up of patient compliance and treatment objectives. Simultaneously, they identified four problems in current multidisciplinary collaboration: 1) the communication of a HF diagnosis to the GP and patient, 2) titration of HF medication by the GP, 3) advanced care planning and 4) electronic data exchange. To address these obstacles and improve current HF care, they proposed increased funding for HF nurses, structured care pathways for HF and a further facilitation and integration of data exchanges.

These findings can guide future implementation of multidisciplinary HF care. The benefits of multidisciplinary team approaches have been amply demonstrated and incorporated into current guidelines [3–5, 17, 18]. However, guidelines provide little detail as to what constitutes multidisciplinary care, nor do they specify which disciplines or intervention domains it should focus. In recent years, increased investment in multidisciplinary care in the community has proved successful [19–21], leading to calls for new multidisciplinary care paradigms based in the home setting in cooperation with primary care [22, 23]. The HF cardiologist and specialist nurse as well as the GP have therefore been defined as the essential triad of any multidisciplinary approach [24], a finding supported by our results. In addition, the dual role ascribed to GPs reflects a consensus among GPs and cardiologists regarding the responsibilities of each discipline [8].

The problems and solutions identified in our study can be contextualized within three disease management taxonomy domains proposed by Krumholz et al. [25], namely methods of communication, delivery personnel and disease management complexity. In the first of these domains, methods of communication, several flawed transfers of information between different health care actors were identified, perhaps most notably to the GP and patient. GPs previously noted the lack of clear communication by cardiologists on the diagnosis of HF [8], illustrated by the use of obfuscating jargon such as “dilative cardiomyopathy” noted by one cardiologist in our paper. Moreover, as reported previously, communication to the patient on HF remains problematic. Although most cardiologists find a discussion of the prognosis in HF important, the majority is reluctant to broach the topic at the time of diagnosis [26]. In addition, a multicontinental cross-sectional survey of more than two thousand patient-cardiologist pairs indicated that they often understate the severity of HF and neglect to discuss treatment choice with almost a third of patients [27]. This reluctance to discuss the diagnosis and prognosis of HF with patients likely contributes to a notoriously low sensitivity of both self-reported HF and concordance of patient awareness with physicians’ assessment of disease severity [28–30].

This could lead to the second and third issues identified in our study. The vague expression of a HF diagnosis and corresponding treatment objectives to GPs could be one of the major drivers behind the suboptimal titration to target dosage of HF medications in primary care experienced by cardiologists in our study, which is a widely recognized issue [31]. A promising remedy would be to relay GPs’ preferences regarding clear and structured discharge reports and implement electronic tools in cardiologists’ EHR that facilitate their generation [32]. In addition, the lack of structured advanced care planning (ACP) in HF is likely driven by physicians’ reluctance to discuss the implications of HF with patients: GPs are hesitant to discuss the topic since patients’ lack awareness of their diagnosis and prognosis and do not often initiate discussions about ACP themselves [33]. Since patients and caregivers with a good understanding of the diagnosis and disease course initiate ACP more frequently, it is certainly probable that increased patient and caregiver education and empowerment would increase the frequency and quality of ACP discussions [34].

Finally, communication issues are exacerbated by impaired electronic data exchange, stemming from the current health care ecosystem in Belgian, with multiple EHR providers and communication platforms operating in parallel without semantic interoperability [35, 36]. Participating cardiologists stressed the need for uniform data approaches, accessible to digitally illiterate patients and facilitating rapid diffusion of relevant information to physicians. These have shown substantial promise in several integrated care projects [37–39].

In addition, two interventions were suggested. First, in the disease management domain of delivery personnel, an extended role for HF nurses. This specialist role is currently unrecognized and unfunded in most European countries [40], leaving many peripheral hospitals without someone to provide basic heart failure education even after a HF hospital admission. In addition, cardiologists suggested a role for HF nurses in primary care, reflecting international trends toward near-home care for HF [20, 23]. HF nurses play an important role in patient communication, in particular explaining the diagnosis and helping patients to understand the condition, because of their long-term relationship with patients and families [7]. Moreover, they facilitate multidisciplinary team communication and promote HF knowledge in other primary care providers. The implementation of such a role is highly dependent on the structure of local and regional care systems. In Belgium, for example, GPs expressed a preference for all-round general practice nurses capable of managing several chronic diseases rather than a variety of primary care clinic specialists [8]. A possible translation of the primary care HF nurse could therefore follow the model of diabetes educators. These educators, i.e. general practice nurses with a special training in diabetes care, instruct patients included in a care trajectory and have improved quality of care processes [41].

Second, in the domain of disease management complexity and intensity a structured approach to interdisciplinary care as exemplified by DMPs or care pathways. These would codify existing agreements between all local and regional health care actors and delineate responsibilities and communication protocols. However, cardiologists echoed the need for patient-centered and flexible trajectories, rather than rigid centralized approaches with disproportionate administrative burdens [8].

### Strengths and limitations

This study has several strengths. First, to our knowledge, this is the first qualitative study to investigate the unique perspective of cardiologists on current and future multidisciplinary collaboration in HF care. Second, we conducted purposive sampling in the region of Limburg, Flanders, which houses two large tertiary hospitals as well as several smaller community hospitals. Therefore, we were able to recruit cardiologists from different backgrounds. Third, we used the robust QUAGOL method to guide data analysis through continuous discussions among the participating authors, thus capturing the essence of each interview before starting the actual coding process.

We acknowledge the limitations of this study. The interviewers worked as GP trainees in the region of Limburg, which could positively bias cardiologists’ responses. Only 12 out of 41 cardiologists agreed to participate, a rather low degree of participation, although we achieved data saturation. However, a thorough training in semi-structured interview techniques and qualitative data analysis compensated for this, as well as the use of investigator triangulation.

## Conclusion

Cardiologists ascribe great importance to close cooperation with GPs and HF nurses in HF care. They identified several problems in current HF care, which are all linked to flawed communication, and proposed an expanded role for HF nurses, integrated data approaches and flexible disease management programs as possible solutions.

## Supplementary Information

**Additional file 1:.** Topic guide.

## Data Availability

The datasets used and/or analyzed during the current study are available from the corresponding author on reasonable request.

## Abbreviations

HF: Heart failure
QUAGOL: Qualitative Analysis Guide of Leuven
DMP: Disease management program
GP: General practitioner
COREQ: Consolidated criteria for reporting qualitative research
HER: Electronic health record
CME: Continuing medical education
