# Supplementary material for: Cardiologists’ perceptions on multidisciplinary collaboration in heart failure care - a qualitative study
Source: BMC Health Serv Res. 2021 Feb 23;21:170. doi: 10.1186/s12913-021-06179-9 (PMC7901167; doi:10.1186/s12913-021-06179-9)
Supplement: Supplementary file 1 — Additional file 1:. Topic guide. [file 12913_2021_6179_MOESM1_ESM.docx]

**Cardiologists’ Perceptions on Multidisciplinary Collaboration in Heart Failure Care**

**A qualitative study**

Willem Raat^a*^, Miek Smeets^a^, Isolde Vandewal^a^, Lien Broekx^a^, Sanne Peters^ab^, Stefan Janssens^c^, Bert Vaes^a^, Bert Aertgeerts^a^

# Supplemental file 1 – Topic guide

Opening question: Can you recall your last contact with a GP about a patient with HF? How did you experience this collaboration?

**CURRENT CARE**

- How do you experience your collaboration with the GP regarding the diagnosis of HF?
  - How do you see the GPs’ role?
  - How do you see your own role?
  - What goes well?
  - What is difficult?
  - What could be improved?
- How do you experience your collaboration with the GP regarding the treatment and follow-up of HF?
  - How do you see the GPs’ role?
  - How do you see your own role?
  - What goes well?
  - What is difficult?
  - What could be improved?

**FUTURE CARE**

- How do you regard future collaboration with GPs around patients with HF?
  - What are the main challenges?
  - What are the main opportunities?
- What is the ‘ideal role’ for cardiologists in this future perspective?
  - What is the ‘ideal role’ for GPs in this future perspective?
  - Who holds the central role?
- What do you think about a care pathway for HF, like the existing ones in Belgium for chronic renal insufficiency and diabetes?
  - How should this be shaped?
  - What barriers do you foresee?
  - What facilitating factors do you foresee?
- What role do you see for HF nurses in the treatment and follow-up of patients with HF?
